# Supplementary material for: Impacts of social isolation and risk perception on social networking intensity among university students during covid-19
Source: PLoS One. 2023 Apr 28;18(4):e0283997. doi: 10.1371/journal.pone.0283997 (PMC10146541; doi:10.1371/journal.pone.0283997)
Supplement: S1 Appendix — (DOCX) [file pone.0283997.s001.docx]

# Appendix A

**Table A1.** List of Constructs and Items

| **Construct** | **Item** | **Mean** |
| --- | --- | --- |
| Social Networking Intensity  [1] | SNI1 | During social distancing/lockdown, I use social networking (SN) more frequently than usual. |
|  | SNI2 | During social distancing/lockdown, I am logging into my SN sites more frequently. |
|  | SNI3 | During social distancing/lockdown, I am messaging my friends more frequently than usual. |
| Subjective  Norms  [2] | SNO1 | Most people who are important to me believe it is adequate for me to take social measures against COVID-19. |
|  | SNO2 | Most people who are important to me understand that I participate social measures against COVID-19. |
|  | SNO3 | Most people who are important to me agree with me about participating in social measures against COVID-19. |
| Perceived Behavioral Control  [2] | PBC1 | I am capable of participating in social measures against COVID-19. |
|  | PBC2 | I am confident that if I want to, I can participate in social measures against COVID-19. |
|  | PBC3 | I have enough resources, time, and opportunities to participate in social measures against COVID-19. |
| Affective Risk Perception  [3] | ARP1 | I'm concerned that I'll get COVID-19. |
|  | ARP2 | Concerned that members of my family may contract COVID-19. |
|  | ARP3 | Concerning the possibility of COVID-19 in my area. |
| Cognitive Risk Perception  [3] | CRP1 | Compared to other diseases, COVID-19 has a high risk of being contracted. |
|  | CRP2 | The chance of dying from COVID-19 is very high. |
| Cabin Fever Syndrome  [4, 5] | CFS1 | Staying at home makes me restless. |
|  | CFS2 | I have problems focusing while I'm at home during social distancing/ lockdown. |
|  | CFS3 | While at home during social distancing/lockdown, I experience social isolation. |

**References**

1. Eid MI, Al-Jabri IM. Social networking, knowledge sharing, and student learning: The case of university students. Computers & Education. 2016;99:14-27.

2. Ajzen I. The theory of planned behavior. Organizational Behavior and Human Decision Processes. 1991;50(2):179-211. doi: 10.1016/0749-5978(91)90020-T.

3. Brug J, Aro AR, Oenema A, De Zwart O, Richardus JH, Bishop GD. SARS risk perception, knowledge, precautions, and information sources, the Netherlands. Emerging Infect Dis. 2004;10(8):1486-9. doi: 10.3201%2Feid1008.040283.

4. Robinson S. Coronavirus Self-Isolation: A Psychologist Explains How To Avoid Cabin Fever, Available at <https://theconversation.com/coronavirus-self-isolation-a-psychologist-explains-how-to-avoid-cabin-fever-133317> (accessed 16 August 2021). 2020.

5. Fritscher L. How to Know If You Have Cabin Fever or Fear of Isolation, Available at: <https://www.verywellmind.com/cabin-fever-fear-of-isolation-2671734> (accessed on 8 June 2021). 2020.
